# A case report: Dual-lead deep brain stimulation of the posterior subthalamic area and the thalamus was effective for Holmes tremor after unsuccessful focused ultrasound thalamotomy

**DOI:** 10.3389/fnhum.2022.1065459

**Published:** 2022-12-15

**Authors:** Satoshi Maesawa, Jun Torii, Daisuke Nakatsubo, Hiroshi Noda, Manabu Mutoh, Yoshiki Ito, Tomotaka Ishizaki, Takashi Tsuboi, Masashi Suzuki, Takafumi Tanei, Masahisa Katsuno, Ryuta Saito

**Affiliations:** ^1^Department of Neurosurgery, Nagoya University Graduate School of Medicine, Nagoya, Japan; ^2^The Center for Focused Ultrasound Therapy, Nagoya Kyoritsu Hospital, Nagoya, Japan; ^3^Department of Neurosurgery, Iwakura Hospital, Iwakura, Japan; ^4^Department of Neurology, Nagoya University Graduate School of Medicine, Nagoya, Japan

**Keywords:** Holmes tremor, deep brain stimulation, thalamus, posterior subthalamic area, dual-lead

## Abstract

Holmes tremor is a symptomatic tremor that develops secondary to central nervous system disorders. Stereotactic neuromodulation is considered when the tremors are intractable. Targeting the ventral intermediate nucleus (Vim) is common; however, the outcome is often unsatisfactory, and the posterior subthalamic area (PSA) is expected as alternative target. In this study, we report the case of a patient with intractable Holmes tremor who underwent dual-lead deep brain stimulation (DBS) to stimulate multiple locations in the PSA and thalamus. The patient was a 77-year-old female who complained of severe tremor in her left upper extremity that developed one year after her right thalamic infarction. Vim-thalamotomy using focused ultrasound therapy (FUS) was initially performed but failed to control tremor. Subsequently, we performed DBS using two leads to stimulate four different structures. Accordingly, one lead was implanted with the aim of targeting the ventral oralis nucleus (Vo)/zona incerta (Zi), and the other with the aim of targeting the Vim/prelemniscal radiation (Raprl). Electrode stimulation revealed that Raprl and Zi had obvious effects. Postoperatively, the patient achieved good tremor control without any side effects, which was maintained for two years. Considering that she demonstrated resting, postural, and intention/action tremor, and Vim-thalamotomy by FUS was insufficient for tremor control, complicated pathogenesis was presumed in her symptoms including both the cerebellothalamic and the pallidothalamic pathways. Using the dual-lead DBS technique, we have more choices to adjust the stimulation at multiple sites, where different functional networks are connected. Intractable tremors, such as Holmes tremor, may have complicated pathology, therefore, modulating multiple pathological networks is necessary. We suggest that the dual-lead DBS (Vo/Raprl and Vim/Zi) presented here is safe, technically feasible, and possibly effective for the control of Holmes tremor.

## 1. Introduction

Holmes tremor is characterized as a symptomatic tremor, which develops secondary to central nervous system (CNS) diseases, including strokes, infections, tumors, trauma, and surgery (Hey et al., 2022). The consensus statement of movement disorder society defined Holmes tremor as resting, postural, and intentional tremor with low frequency and irregular amplitude (Krauss and Jankovic, 2002; Bhatia et al., 2018). Although the pathogenesis remains unclear, combined abnormalities including the dopaminergic nigrostriatal system, cerebello-thalamo-cortical pathway, and dentate-rubro-olivary pathway are considered to be associated with tremor development (Vidailhet et al., 1998; Akkus and Diramali, 2006).

Raina et al. (2016) reported that cerebrovascular disease (ischemia or hemorrhage) was the most common cause of Holmes tremor among the CNS diseases (48.3%). This symptom generally appeared late, and the median interval between the onset of CNS disease and tremor was reported to be 2 months (range, 7 days to 228 months). These lesions involve various subcortical structures, including the midbrain, thalamus, pons, and cerebellum. Some patients with Holmes tremors respond to L-dopa and dopamine agonists (Velez et al., 2002; Akkus and Diramali, 2006), whereas others are often medically refractory. In these cases, neuromodulation by stereotactic and functional techniques is considered, including deep brain stimulation (DBS), radiofrequency ablation (RF), and magnetic resonance-guided focused ultrasound ablation (MRgFUS). The ventral intermediate nucleus (Vim) is a common target site. Approximately two decades ago, the posterior subthalamic area (PSA) was also shown to be effective for the control of essential tremors and other intractable tremors (Velasco et al., 2001; Murata et al., 2003; Plaha et al., 2008; Fytagoridis et al., 2012). However, patients with Holmes tremor is often more intractable than in those with essential tremor.

We assume that neuromodulation of multiple targets is necessary to control such intractable tremors. Here, we report the case of a patient with intractable Holmes tremor, for whom MRgFUS targeting at the Vim was initially performed, but failed in tremor control, and thereafter dual-lead DBS targeting the PSA in addition to the thalamus was performed with implantation of two leads. The targeting sites were established to modify four different structures, the Vim to the prelemniscal radiation (Raprl) and the ventral oralis (Vo) to the zona incerta (Zi). The patient achieved good postoperative tremor control. Although several techniques for dual-lead DBS have been reported, to the best of our knowledge, this specific method has never been reported. Here, we present the techniques and outcomes of this surgery and discuss its clinical importance in intractable tremors.

## 2. Case presentation

### 2.1. History of present illness

**Figure 1:**
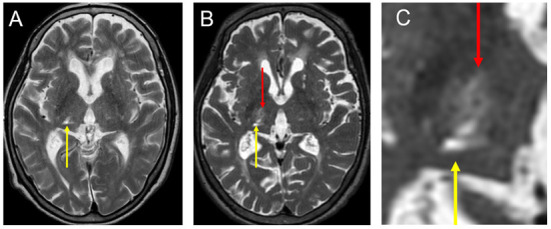
Preoperative images of her MRI. **(A)** T2 image taking before FUS—thalamotomy after right thalamic infarction (yellow arrow). The infarction was located in the right VC extending to the pulvinar nucleus. **(B)** T2 image taking a day after FUS—thalamotomy. Note that the lesion with super low intensity with surrounding high intensity was located in the Vim (red arrow). **(C)** High magnification of B. FUS, focused ultrasound surgery; MRI, magnetic resonance imaging; VC, ventral caudalis nucleus; Vim, ventral intermediate nucleus.

We present the case of a right-handed 77–year-old female patient who developed a right thalamic infarction at the age of 74 years (Figure 1A). She developed numbness in the left upper and lower extremities, which gradually improved but did not completely dissipate. Approximately 1year after the onset of the infarction, she developed a tremor in her left upper extremity, which gradually worsened. She had no family history associated with tremor, and no medical history of diseases associated with tremor, such as hyperthyroidism. She was diagnosed with Holmes tremor and was subsequently treated with atenolol hydrochloride, L-dopa, and clonazepam. However, this combination therapy failed to control her tremor. Two years after the onset of the infarction, she was referred to our hospital for surgical intervention.

### 2.2. Preoperative evaluation

The patient had a rough and severe tremor in the left upper extremity, which was characterized by a frequency of approximately 3 Hz, and appeared in arm-stretching or wing-beating posture and goal-directed movement. Resting tremors were also observed. Dystonic postures and movements were accompanied by tremors, especially when writing letters. Additionally, she had mild and chronic numbness in her left upper and lower extremities, without other neurological deficits. Parkinsonism was not observed. Her cognition was within normal limits. Magnetic resonance imaging (MRI) revealed a small, old infarction in the ventral-posterolateral area of the thalamus, which was mainly consistent with the ventral caudalis (VC) and the pulvinar nucleus (Figure 1A). A dopamine transporter (DaT) scan showed a normal pattern. The skull density ratio (SDR) was 0.33. The Clinical Rating Scale for Tremor (CRST) score, which indicates tremor severity, was preoperatively examined. Briefly, the CRST part A score is related to tremors in nine parts of the body, whereas the CRST part B score is related to tremors that occur while writing and pouring liquids. The CRST part C score was used to assess the patients' quality of daily life. Her total CRST score was 46: 16 for part A; 16, for part B, and 14 for part C. The CRST in the diseased upper extremity (left) was 25, 9 for part A, and 16 for part B.

### 2.3. Surgical interventions

Three stereotactic options (DBS, RF, and MRgFUS) were presented, and the patient and her family were sufficiently informed about the general indications, risks, and benefits of the respective procedures. Although she understood that her SDR was less than optimal and the effectiveness of MRgFUS for Holmes tremor was not well established, she chose Vim-thalamotomy by MRgFUS because of the non-invasiveness of this treatment. MRgFUS was performed at the age of 76 (two and a half years after the onset of infarction) and showed no adverse events except for headache and nausea during the procedure. The maximum average temperature was 54°C, and the location of the lesion was considered appropriate (Figures 1B, C). Although she demonstrated slight improvement immediately after MRgFUS, her symptoms recurred at a follow-up of 1 month. The patient required another surgical intervention.

**Figure 2:**
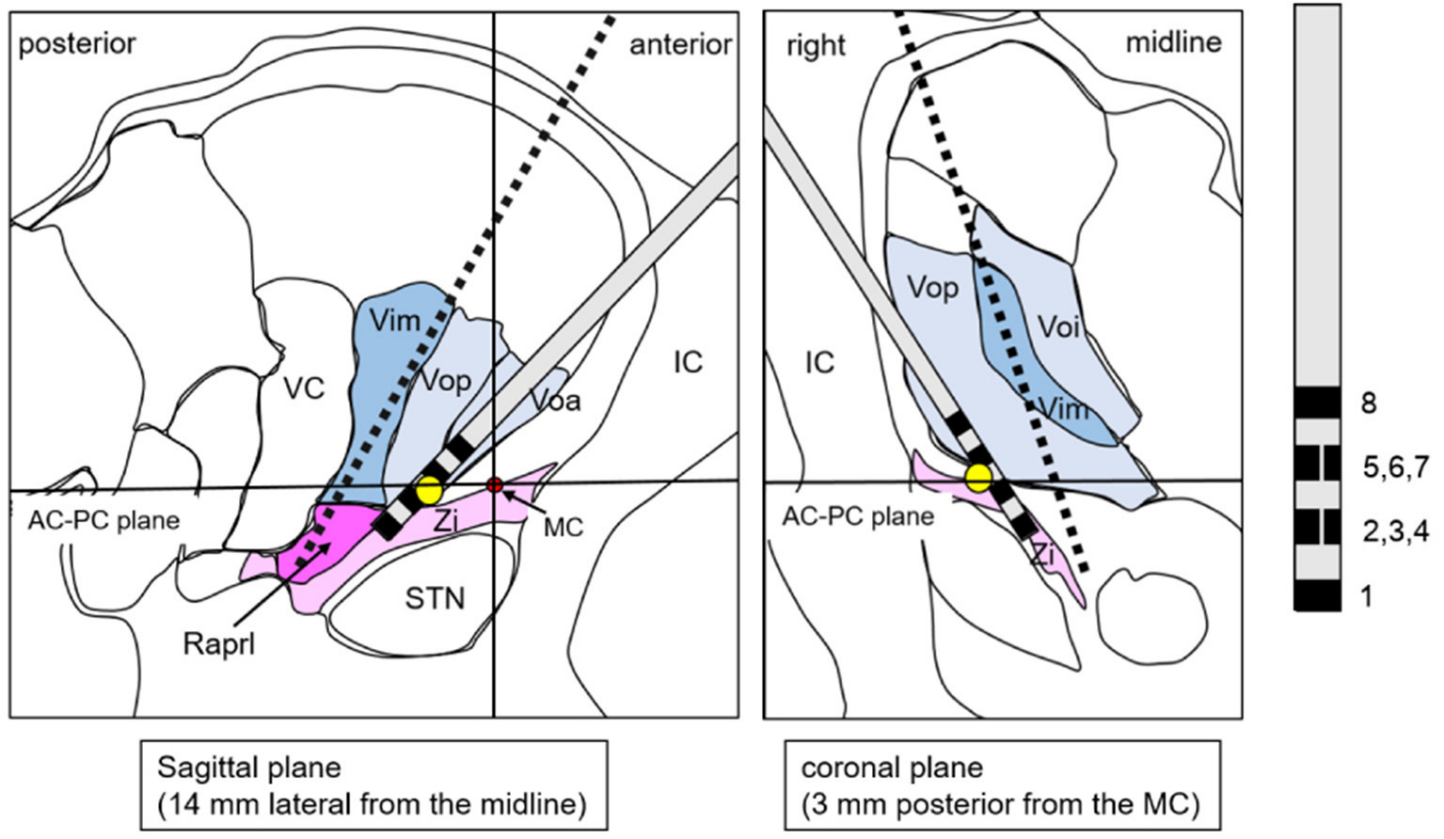
A schema shows the lead targeting the Vo/Zi superimposed on the atlas, which is adopted from the stereotaxy atlas (14). The yellow circle represents the initial target. Red circle represents the location of the MC. Black broken line is transparent image of the other lead. AC, anterior commissure; IC, internal capsule; MC, midpoint of AC-PC line; PC, posterior commissure; Raprl, prelemniscal radiation; STN, subthalamic nucleus; VC, ventral caudalis; Vop (a, i), ventral oralis nucleus posterior (anterior, interna); Zi, zona incerta.

**Figure 3:**
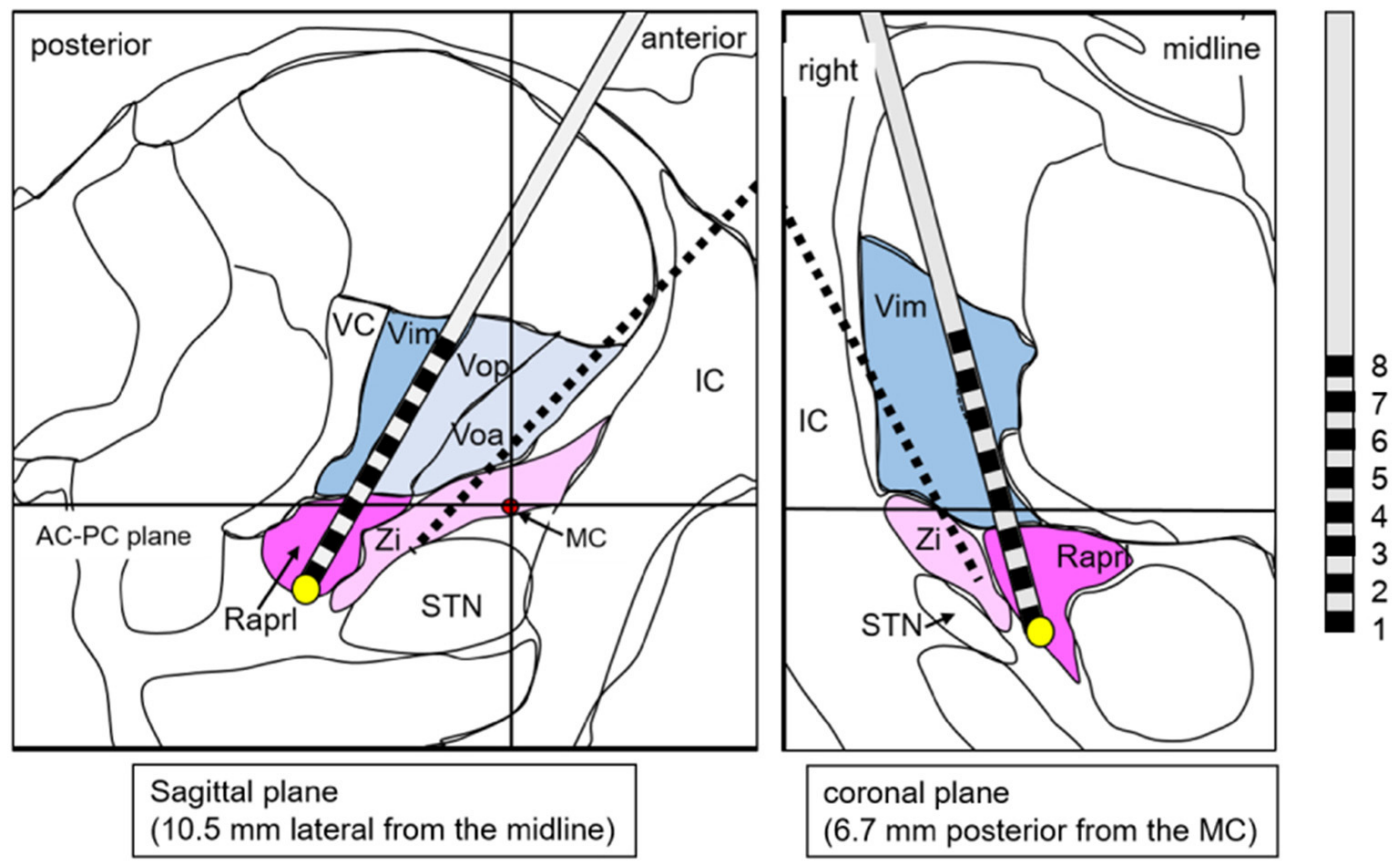
A schema shows the lead targeting the Vim/Raprl superimposed on the atlas, which is adopted from the stereotaxy atlas (Schaltenbrand and Wahren, 1977). The yellow circle is the initial target. Red circle is the location of the MC. Black broken line is transparent image of the other lead. AC, anterior commissure; IC, internal capsule; MC, midpoint of AC-PC line; PC, posterior commissure; Raprl, prelemniscal radiation; STN, subthalamic nucleus; VC, ventral caudalis; Vop (a), ventral oralis nucleus posterior (anterior); Zi, zona incerta.

Based on the fact that a lesion has already been created in the Vim but failed to control tremor by MRgFUS, we suggested DBS dually targeting the PSA in addition to the thalamus. Considering the possibility that stimulation of the Vo may also be effective based on reports from other facilities (Foote and Okun, 2005; Gallay et al., 2008; Oliveria et al., 2017), we also included Vo as a target. Accordingly, we planned dual-lead DBS; one target was the Vo + Zi (Figure 2), and the other target was Vim + Raprl (Figure 3). The lead for the Vo and the Zi was placed laterally and anteriorly, and the initial target coordination was calculated with reference to the Schaltebrand atlas (Schaltenbrand and Wahren, 1977). The X coordinate was 14.0 mm right from the midline, the Y coordinate was 3.0 mm posterior from the midpoint of the anterior-posterior commissure (AC-PC) line, and the Z coordinate was just at the AC-PC plane. The initial target was set at the border of the thalamus and Zi, and we intended to insert it deeper from the target with microelectrode recording guidance. The angle to the AC-PC plane was 46° and the angle from the midline was 26° (Figure 2). On the other hand, the other lead for the Vim and the Raprl was placed medially and posteriorly, the initial target was set at the bottom of the Raprl; X coordinate was 10.5 mm right from the midline, Y coordinate was 6.7 mm posterior from the midpoint of AC-PC line, and Z coordinate was 4 mm inferior from the AC-PC plane. The angle to the AC-PC plane was 65°, and the angle from the midline was 19° (Figure 3). Surgical planning was performed using a planning workstation, Voxim (Renishaw, New Mills, UK) (Supplementary Figures S1A–D). During the surgical procedure, the cranial part was subjected to local anesthesia. A Leksell stereotactic frame (Elekta, Stockholm, Sweden) was used, and electrodes were inserted using Neuromate (Renishaw, New Mills, UK). The microelectrode recording (MER) was performed. We found irregular and hyperactive activity in the Vim, with some sensorimotor responses in the upper limb, but the neuronal activities in the Raprl were not identifiable from the background. We also found some neuronal activity in the Vo, but the activity in the Zi was not obvious. However, 3–5 mm deep from the presumed location of the Zi, we obtained hyper-neuronal activity, consistent with the subthalamic nucleus (STN). Macro-stimulation of 1–3 mA was performed at these four targets, and no side effects were confirmed. Finally, two stimulation leads were implanted. In this case, the directional lead, which consisted of the deepest ring (contact 1), the second deepest ring with three different directed parts (contacts 2, 3, and 4), the third ring with three different directed parts (contacts 5, 6, and 7), and the superficial ring (contact 8) (Boston Scientific, USA), was utilized for the electrode targeting at Vo + Zi. This was because we attempted to prevent anterior stimulation of the internal capsule by using its directivity. The contacts 1 to 4 were in the Zi, and 5 to 8 were in the Vo (Figure 2). For the Vim + Raprl targeting lead, a twisting lead (Boston Scientific, USA), which has eight contacts (contacts 1 to 8) and enables stimulation over a relatively long distance along the axis, was utilized. The contacts 1 to 4 were in the Raprl, and 5 to 8 were in the Vim (Figure 3). For the pulse-generator, Genus P16 (Boston Scientific, USA) was implanted into the subcutaneous space of the left chest under general anesthesia. All the procedures were completed without complications.

### 2.4. Postoperative course

The postoperative course was uneventful, and imaging studies, including radiography (Supplementary Figures S1E, F) and computed tomography, demonstrated the optimal location of leads without complications. Electrostimulation screening revealed that Vim and Vo had weak effects in this case, while Raprl and Zi had obvious effects on the tremor. By performing dual-lead DBS combined with Raprl and Zi stimulation, the tremor suppression effect was greater than that of a single stimulation. Numbness was likely induced by stimulation in the posterior area, where the VC was located, and we assumed some post-infarction effects. Dysarthria was also likely to be induced by stimulation extending to a wide area with high current intensity; therefore, we attempted to limit the stimulated area by bipolar stimulation. Finally, the stimulation conditions were as follows: Raprl: the contact setting was 1 (-) and 2 (+), the intensity was 3.0 mA, duration was 50 μs, and the frequency was 149 Hz; and Zi: the contact setting was 1 (-), 2, 3, and 4 [ring-mode stimulation at the second deepest contacts (+)], the intensity was 3.0 mA, the duration was 50 μs, and the frequency was 149 Hz. The summary of electrostimulation screening is shown in the Table 1. Although the tremor did not completely disappear, she had good tremor control without any complications, and her quality of daily life significantly improved. Postoperative CRST score at the 6-month follow-up totaled 32 (30 % reduction): 6 for part A (62.5% reduction), 13 for part B (18.7 % reduction), and 10 for part C (28.6 % reduction). CRST in the diseased upper extremity (left) totaled 17 (32 % reduction): 4 for part A (55.5% reduction), and 13 for part B (18.7 % reduction). Good tremor control was maintained for 2 years after surgery.

**Table 1:** Summary of electrostimulation screening with dual-lead DBS for Holmes tremor (gray background shows optimal setting in monopolar and bipolar condition).

**Location of stimulation**	**Contact**	**Intensity (mA) /Hz /duration (μsec.)**	**Postural tremor (cm)**	**Action tremor (cm)**	**Adverse effects**
**Monopolar stimulation testing**				
Baseline	N/A	N/A	20	20	N/A
Raprl	1 (-)	3 / 130 / 40	0	4	None (≥3 mA) Dysesthesia and dysarthria (> 3 mA)
	2 (-)		0	2–4	
	3 (-)		2–4	10	
	4 (-)		4	10	
Vim	5 (-)		2–4	10	
	6 (-)		2–4	10	
	7 (-)		4	10	
	8 (-)		2–4	6–10	
Zi	1 (-)	3 /154 / 40	6–10	20	None (≥3 mA) Dysesthesia and dysarthria (> 3m A)
	2,3,4 (-)		6–10	20	
Vo	5,6,7 (-)		20	20	
	8 (-)		20	20	
Raprl + Zi	Raprl = 1 (-) Zi = 1 (-)	2 /149 / 50	2	2	None (< 2.5 mA) Dysesthesia (≥ 2.5 mA)
**Bipolar stimulation testing**					
Raprl + Zi	Raprl =1 (-) 2 (+) Zi = off	2 /149 / 50	0	4–6	None (< 3.5 mA) Dysesthesia (≥ 3.5 m)
	Raprl = 1 (-) 2 (+) Zi = 1 (-) 2,3,4 (+)	2–3 /149/ 50	0	2	
	Raprl = 1 (+) 2 (-) Zi = 1 (+) 2,3,4 (-)	2 / 148 /50	2	4–6	
Raprl/Vo + Zi/Vim	Raprl/Vim = 2 (-) 3 (+) Zi/Vo = 2,3,4 (-) 5,6,7 (+)	2 /149 /50	2	10	None (< 3.5 mA) Dysesthesia (> 3.5 mA)
	Raprl/Vim = 1(-) 2(-) 3 (+) Zi/Vo = 2,3,4 (-) 5,6,7 (+)		2	10	

DBS, deep brain stimulation; μsec, microseconds; N/A, not attribute; Vim, ventral intermediate nucleus; Vo, ventral oralis nucleus; Zi, zona incerta; Raprl, prelemniscal radiation. The gray background setting indicates the monopolar and bipolar condition.

## 3. Discussion

### 3.1. Where is the optimal DBS target for Holmes tremor?

Holmes tremor occurs after various subcortical lesions and presents with complex symptoms. When performing neuromodulatory therapies, it is necessary to consider which functional networks are affected. Mendonça et al. reported a review and meta-analysis of DBS outcomes for Holmes and lesion-related tremors, summarizing 35 papers on 82 patients (Mendonça et al., 2018). They reported that Vim DBS followed by the globus pallidum interna (GPi) DBS were preferred, and tremor improvement after DBS in patients with post-stroke Holmes tremor was 77.5%. Among them, 13.5% of the patients had < 50% tremor improvement. More recently, Wang et al. conducted a review and meta-analysis of tremor outcomes for Holmes tremor, emphasizing that DBS targeting the GPi had greater benefits than the Vim (Wang et al., 2022). Interestingly, there were a few patients successfully treated by dual-lead stimulation with the Vo and other targets such as the PSA. This finding suggests heterogenicity among the patients with Holmes tremor.

Recently, Joutsa et al. performed mapping for Holmes tremor network using Human Brain Connectome (Joutsa et al., 2019), and found that there was a common circuit consisted of nodes in the red nucleus, thalamus (Vo), GPi, and cerebellum. They noted that network disturbance in Holmes tremor may be more complicated compared to that in essential tremor. The pallidothalamic pathway may play an essential role in the pathogenesis of Holmes tremor, and thereby be the preferred surgical targets for patients with Holmes tremor. In our case, Vim-thalamotomy using MRgFUS was ineffective. This result indicates the involvement of other functional networks aside from the cerebellothalamic pathway.

### 3.2. PSA DBS and feasibility of dual-lead implantation

PSA DBS has been shown to be efficacious for patients with intractable tremors, even with DBS or ablation targeting the Vim (Velasco et al., 2001; Murata et al., 2003; Plaha et al., 2008; Fytagoridis et al., 2012; Barbe et al., 2018). The PSA is an anatomical region consisting of Raprl and Zi. The Raprl is a white matter structure that contains the cerebellothalamic tracts [alternatively called as the dentato-rubro-thalamic tract (DRTT)] and mesencephalic reticular thalamic tracts, while the Zi relays the cerebellar-thalamic-cortical and pallidothalamic pathways (Watson et al., 2014). While both are known to be effective in suppressing tremors, possible side effects, such as balance dysfunction, ataxia, dysarthria, and diplopia, are likely to be induced by relatively lower stimulation than the Vim (Barbe et al., 2018; Hidding et al., 2019). The PSA covers a fairly wide area, and the optimal target for tremor control with minimal adverse effects differs among studies (Velasco et al., 2001; Murata et al., 2003; Plaha et al., 2008; Fytagoridis et al., 2012; Barbe et al., 2018; Hidding et al., 2019). As described above, the Zi and Raprl play different roles in functional networks, and their locations are geographically distant. A large volume of tissue activated covering both structures using a single lead may readily cause adverse effects. Therefore, we decided to place two electrodes independently in the Zi and Raprl to stimulate both structures with minimal risk of adverse effects.

In previous reports, various methods have been introduced for dual-targeting DBS, including Raprl following Vim, Raprl + Zi following Vim (Kobayashi et al., 2014; Bot et al., 2018; Dos Santos Ghilardi et al., 2018), and PSA + STN following Vim (Coenen et al., 2016; Neudorfer et al., 2019). Notably, simultaneous stimulation of the Vim/Raprl and the Vo/Zi using two leads, which we employed for our case, was also described as a theoretically feasible model (Iorio-Morin et al., 2020). However, there have been no reports about actual clinical use.

The current DBS systems enable stimulation adjustment of two leads using a single pulse-generator device. Therefore, in unilateral Holmes tremor, multiple targeting methods using dual electrodes may be technically feasible and more effective, as in the present case. Patients with Holmes tremor may have complicated pathology affecting multiple functional networks, including the cerebellothalamic pathway and the pallidothalamic pathway. Therefore, stimulation using dual electrodes can modify these networks, which may lead to better outcomes. In our case, electrodes were placed dorsally in the Vim and the Vo separately, and ventrally in both the Zi and Raprl in the PSA. A single tract could anatomically cover both the Vo and Zi or both the Vo and Raprl; therefore, this method is technically reasonable.

### 3.3. Future perspective and limitations

As future steps to validate the effectiveness of our dual-lead DBS technique, imaging-based studies on multiple cases should be conducted. More specifically, tractography methods, visualizing the Raprl (Lefranc et al., 2015), DRTT (Fenoy and Schiess, 2018), and other tracts, may shed more light on the pathogenesis of Holmes tremor. In addition, the effects of different stimulation settings, such as amplitude, stimulation frequency, and pulse width, should be tested (Hidding et al., 2022). These studies may contribute to explain our hypothesis that Holmes tremor involve multiple networks.

Our case study had the following limitations. First, the stimulation of the Vim and Vo was ineffective for tremor control, which indicated that the contacts may not be optimally located in these structures. The lead for Vim/Raprl could have been more effective if it passed more laterally in the Vim. Second, we did not use stages approach in this case, which could be a good option as it is less invasive. Thirdly, it should be noted that the more electrodes implanted, the greater the risk of intracranial hemorrhage or postoperative infection.

## 4. Conclusion

In conclusion, our method using dual electrodes could potentially provide us with more options to adjust the stimulation at multiple regions. As a result, we could achieve optimal tremor control while minimizing potential adverse effects. We confidently suggest that dual-targeting DBS for Vo/Zi and Vim/Raprl is a feasible and effective method for patients with intractable tremors who have complicated pathologies.

## Data availability statement

The original contributions presented in the study are included in the article/Supplementary material, further inquiries can be directed to the corresponding author.

## Ethics statement

The studies involving human participants were reviewed and approved by the Local Ethics Committee of Nagoya University (No. 2017-0147). The patients/participants provided their written informed consent to participate in this study.

## Author contributions

Substantial contributions to the conception and design of the work: SM, JT, DN, TTs, MK, and RS. Acquisition and analysis of the data: SM, JT, DN, MM, HN, YI, TI, MS, and TTa. Interpretation of data: SM, DN, TTs, MK, and RS. Drafting the work and revising it critically for important intellectual content: SM and JT. All authors approved the final version of the article.

## Supplementary material

Supplementary Figure S1 Surgical plan of the dual-lead DBS; the Vo/Zi, and the Vim/Raprl. **(A)** Axial, **(B)** Coronal, **(C)** Sagittal image: The lead for the Vo/Zi shows green line, and the other for the Vim / Raprl shows yellow line. **(D)** An overview of two leads (the Vo/ZI: red, the Vim/Raprl: yellow). **(E,F)** Were postoperative X–ray images showing two-lead DBS. AC, anterior commissure; MC, midpoint of AC-PC line; PC, posterior commissure; Raprl, prelemniscal radiation; Vim, ventral intermediate nucleus; Vo, ventral oralis nucleus; Zi, zona incerta.
