# Supplementary figures and images for: A case report: Dual-lead deep brain stimulation of the posterior subthalamic area and the thalamus was effective for Holmes tremor after unsuccessful focused ultrasound thalamotomy
Source: Front Hum Neurosci. 2022 Dec 15;16:1065459. doi: 10.3389/fnhum.2022.1065459 (PMC9798537; doi:10.3389/fnhum.2022.1065459)

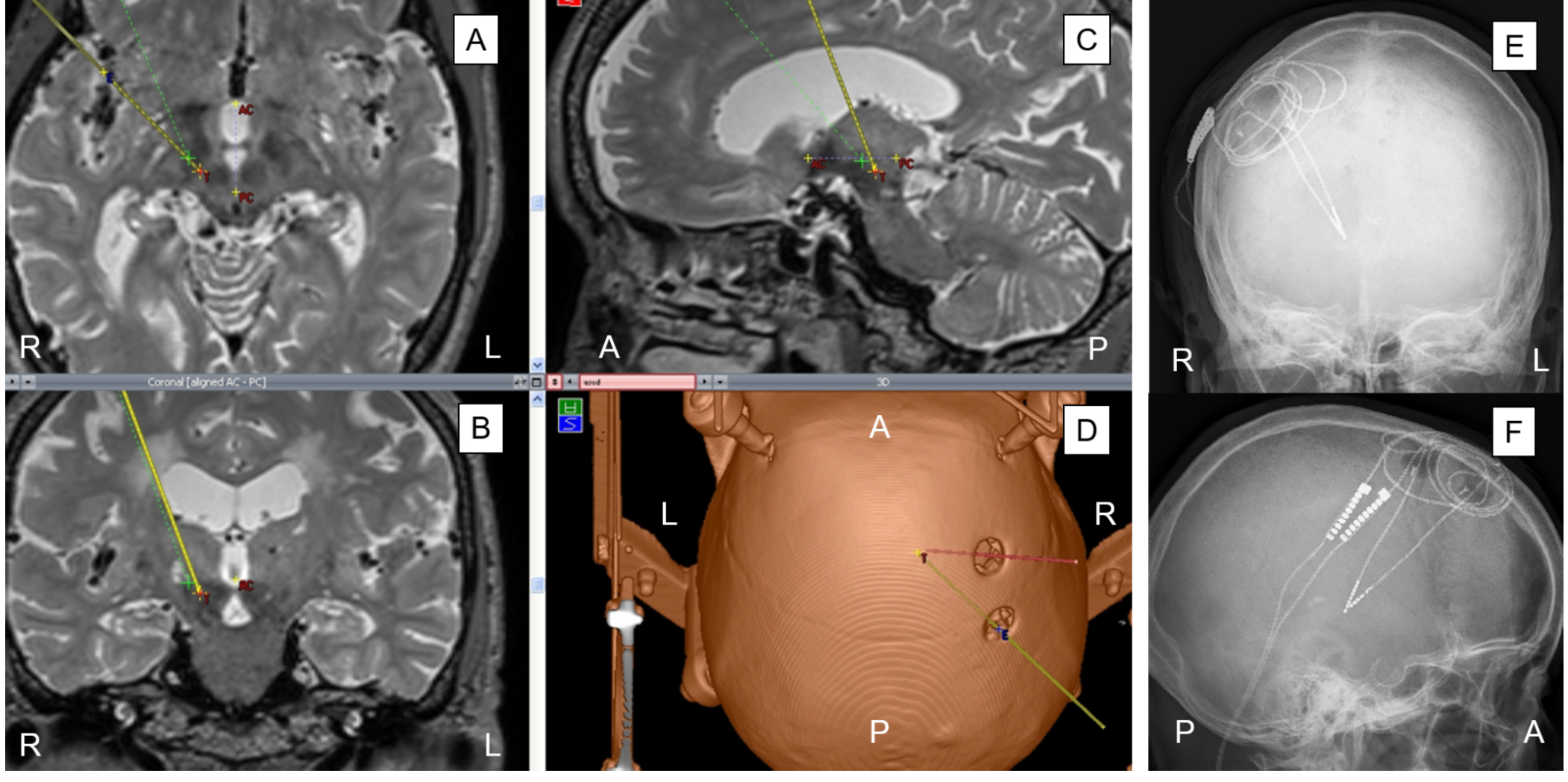

Supplement: Supplementary Figure S1 — Surgical plan of the dual-lead DBS; the Vo/Zi, and the Vim/Raprl. (A) Axial, (B) Coronal, (C) Sagittal image: The lead for the Vo/Zi shows green line, and the other for the Vim / Raprl shows yellow line. (D) An overview of two leads (the Vo/ZI: red, the Vim/Raprl: yellow). (E,F) Were postoperative X–ray images showing two-lead DBS. AC, anterior commissure; MC, midpoint of AC-PC line; PC, posterior commissure; Raprl, prelemniscal radiation; Vim, ventral intermediate nucleus; Vo, ventral oralis nucleus; Zi, zona incerta. [file Image_1.JPEG]
